# The learning curve for a single surgeon using ultrasonography to guide supine percutaneous nephrolithotomy with an alken metal telescopic dilator

**DOI:** 10.1016/j.heliyon.2022.e12524

**Published:** 2022-12-21

**Authors:** Ponco Birowo, Reginald Rustandi, Putu Angga Risky Raharja, Harun Wijanarko Putra, Nur Rasyid, Widi Atmoko

**Affiliations:** Department of Urology, Faculty of Medicine, Universitas Indonesia, Cipto Mangukunsumo Hopital, Jakarta, Indonesia

**Keywords:** Percutaneous nephrolithotomy, Alken metal telescopic dilator, Ultrasound, Learning curve

## Abstract

Ultrasound (US) has three advantages over fluoroscopy for guiding percutaneous nephrolithotomy (PNCL): it provides an assessment of adjacent structures and real-time puncture adjustment, and is radiation free. This study aimed to define the number of procedures that should be performed to achieve competence in US-guided PCNL using an Alken metal telescopic dilator. A non-randomised retrospective study with consecutive sampling was used for the study design. A total of 50 patients above 18 years of age with the largest diameter of renal stone ≥20 mm were included. They were divided into five groups based on timing of the surgery to evaluate and visualise improvements based on primary outcomes within the groups. Line charts were used, and statistical analysis was performed to evaluate the learning curve. Most of the base characteristics between the groups were similar. Tract dilatation time decreased significantly after 20 PCNLs were performed (p < 0.001). Stone-free status markedly increased after 20 PCNLs were performed (p < 0.001). Postoperative fever (10%) and need for blood transfusion (26%) were the only complications. Basic competency was achievable after 20 PCNL procedures were performed, and further improvements in outcomes were achieved after 40 PCNLs with an acceptable rate of non-severe complications.

## 1 Introduction

Since its introduction in 1976, percutaneous nephrolithotomy (PCNL) has become the gold standard treatment for renal stones >2 cm [1, 2]. Deciding which guiding technique to use in obtaining renal access is important. Access to the renal calyx is usually achieved by fluoroscopic evaluation, despite other methods such as ultrasound which would eliminate radiation exposure for both patients and physicians [2].

A patient's radiation exposure from fluoroscopy can be as high as 0.91 mSv for 75 min on average, while for the physicians, it could be as high as 0.41 mSv [3]. The recommended occupational exposure limit for medical personnel is 50 mSv per year, and radiation doses as little as 10 mSv may result in the development of malignancy in 1 of 1,000 individuals exposed [4]. Doctors at Dr. Cipto Mangunkusumo Hospital, a national referral hospital, have performed 1,300 PCNL procedures over 17 years (2000–2017), with an average of 70 procedures per year [5]. Even though the resulting average exposure is within the recommended occupational limits, ionising radiation still poses a risk, especially to those with repeated exposures.

The use of ultrasound would eliminate the radiation risk and provide an effective alternative, primarily for non-opaque stones that are not visible using fluoroscopy [1]. With ultrasound, organs near the puncture site (bowel, liver, spleen, and lung) are easily identified, but this is not possible with fluoroscopy [6]. Therefore, ultrasound has the potential to replace fluoroscopy considering its advantages, despite the expertise needed for it to be correctly performed. In addition to using ultrasound for its guiding, the Alken metal telescopic dilator was used as a tract dilator instead of a costly and non-reusable balloon dilator.

More evidence is needed regarding the time it takes for a surgeon to become trained in using ultrasound instrumentation. Therefore, this study aimed to evaluate the learning curve of a single urologist in competently performing ultrasound-guided (USG) supine PCNL using an Alken metal telescopic dilator.

## 2 Methods

This study used a non-randomised retrospective study design with consecutive sampling of patients who underwent PCNL at a single-site from October 2019 until March 2020. Patients over 18 years old and presenting with a renal stone or an accumulative stone size diameter of more than 20 mm were included there is no randomisation, the data collected through consecutive sampling. The samples included were patients who met the inclusion criteria and does not met the exclusion criteria during the period of the study. Patients with anatomical anomalies, uncorrected coagulopathy prior to surgery, untreated urinary tract infection, tumours in the presumptive access tract area, potential malignant kidney tumours, and those receiving anticoagulant therapy or pregnant, were excluded.

Patients were divided into those who underwent USG supine PCNL (n = 50) as the treatment group and those who underwent supine PCNL using fluoroscopy (n = 10) as the control group. The patients in the treatment group were divided into five subgroups, consisting of ten patients each, according to the timing of surgery. Evaluation of these groups was based on three key outcomes: conversion rate to fluoroscopy, renal access and tract dilatation time, and stone-free rate. A trend line plotting the outcomes was used to visualise improvement over time. The control group served to show a comparison of the stone-free rate and complication rate for the last treatment group. Because the data on PCNL using fluoroscopy were retrospective, the skin-to-stone distance, renal access time, and tract dilatation time in the control group were unavailable.

The study was approved by The Ethics Committee/Institutional Review Board of The Faculty of Medicine, Universitas Indonesia, under ethical approval number 442/UN2.F1/ETIK/PPM.00.02/2020. Informed consent from the patients was waived and approved by The Ethics Committee/Institutional Review Board of The Faculty of Medicine, Universitas Indonesia, under the approval number mentioned due to the retrospective study design where data was taken from medical records and no intervention was performed.

All USG supine PCNLs and fluoroscopy supine PCNL were performed by a single urologist in this study. This was done to prevent bias of variation in individual ability while performing PCNL.

### 2.1 Surgical variables

**Figure 1:**
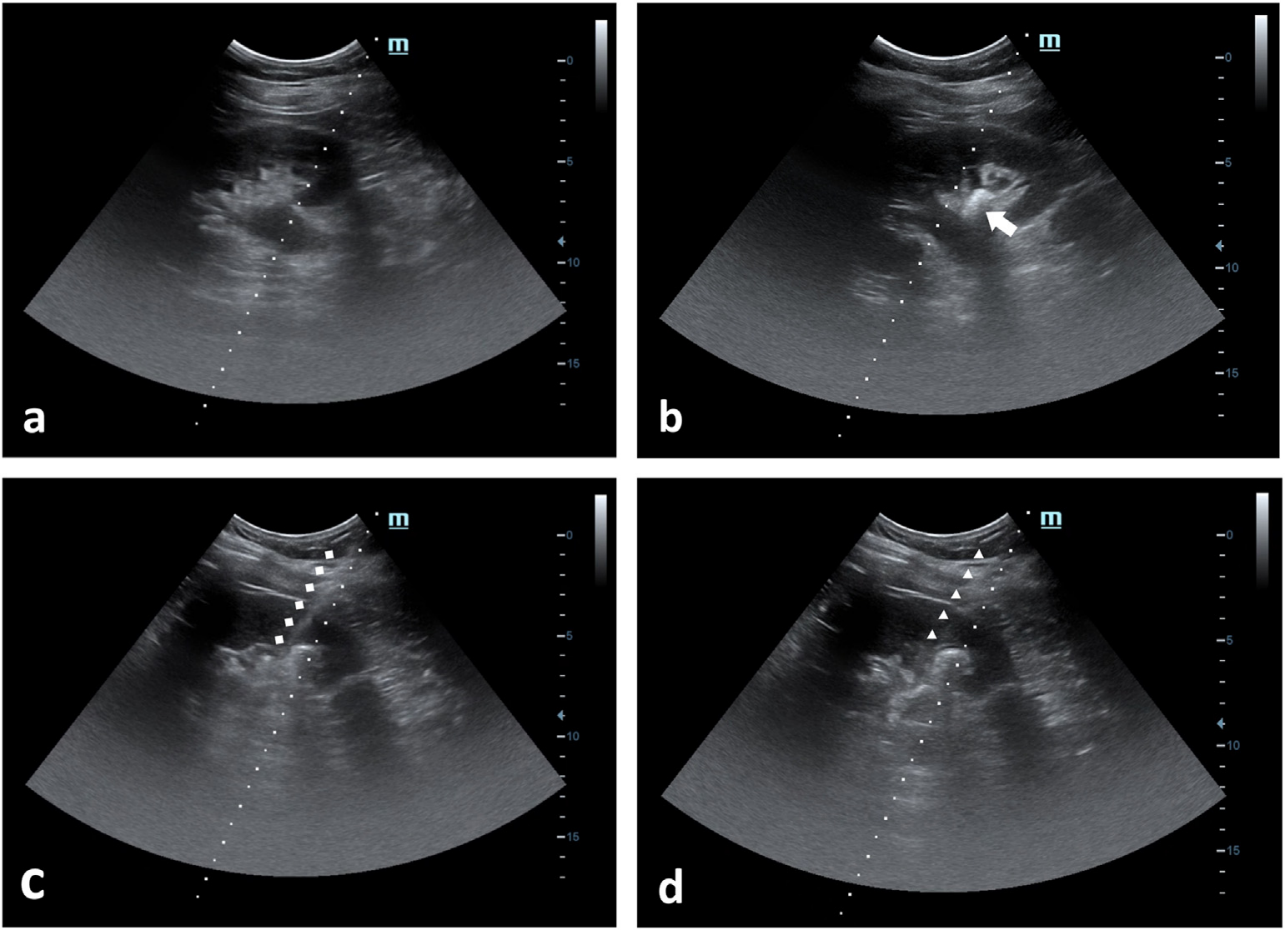
Renal ultrasound in PCNL (a) visualisation of the kidney and its surrounding before ureteral catheter insertion. (b) the tip of the ureteral catheter was seen at the opening of inferior calyx. (c) Needle tract was clearly seen piercing through renal parenchyma, into the pelvic calyceal system. (d) Alken metal dilator can clearly be seen through ultrasound imaging.

**Figure 2:**
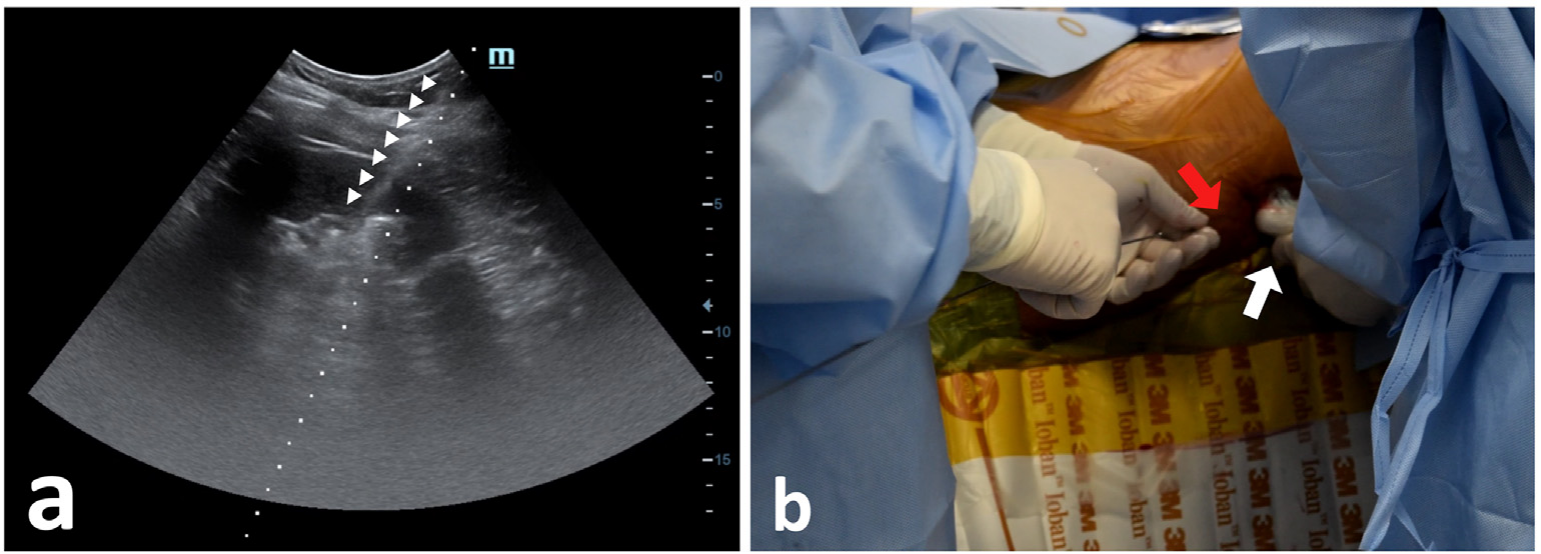
Ultrasound guided renal puncture. (a) Visualisation of Needle tract clearly seen piercing through renal parenchyma, into the pelvic calyceal system. (b) Needle sized 17,5 G, held by operator, puncturing the flank area (red arrow) guided by ultrasound visualization. Ultrasound probe (white arrow) held by the operator assistant.

**Figure 3:**
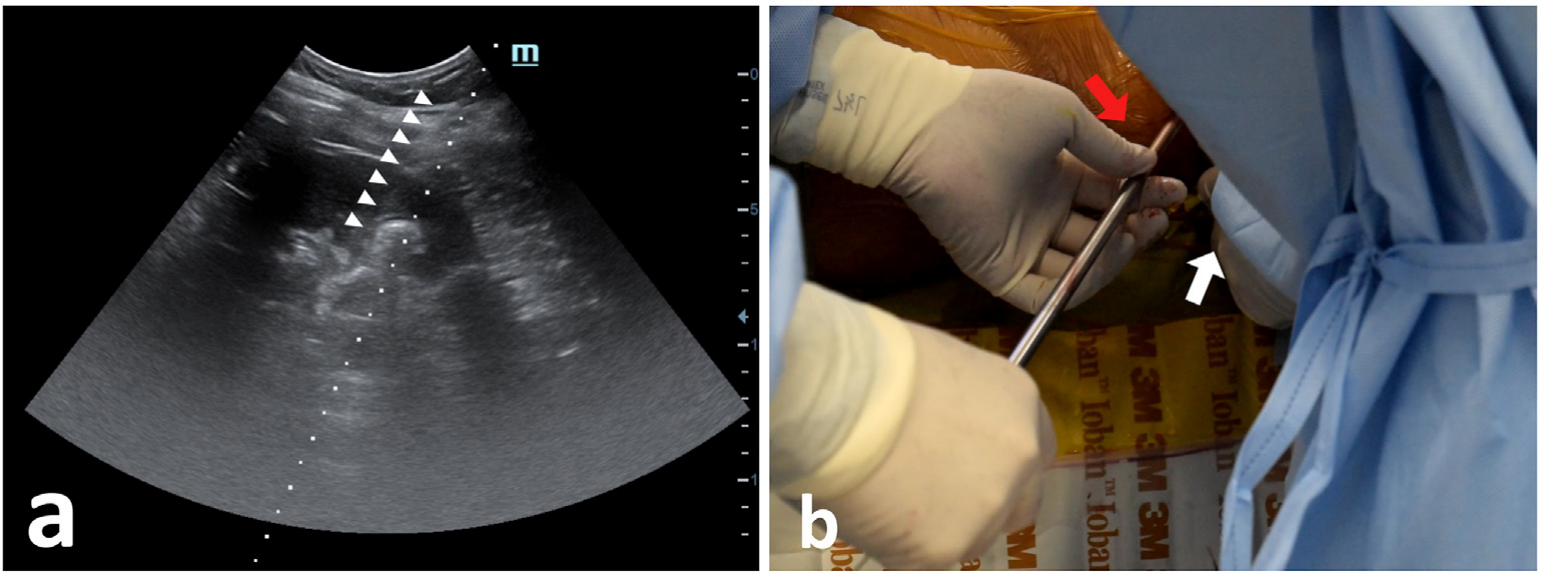
Ultrasound guided renal tract dilatation using alken metal dilator. (a) Visualisation of Alken metal dilator can clearly be seen through ultrasound imaging. (b) Alken metal dilator, held by operator, inserted serially (red arrow) up to 30 Fr guided by ultrasound visualization. Ultrasound probe (white arrow) held by the operator assistant.

Routine blood evaluation, anaesthesia assessment, and non-contrast computed tomography (CT) urography prior to the operation were performed, and on the day of the operation, a prophylactic antibiotic was administered. Spinal anaesthesia was used in all cases included in the study. After spinal anaesthesia is induced, a- and anti-septic procedure were done. Patients were then positioned in modified lithotomy position, where the contralateral lower-limb was in lithotomy position; while the ipsilateral lower-limb was abducted as lateral as possible. Two cushions were placed in the thorax and pelvis ipsilateral to the PCNL side, thus, provide elevation approximately 15° relative to the operating table and cystoscopy was then performed. Upon finished, the ipsilateral lower-limb was then adducted as medial as possible, to provide enough space for puncture, dilatation and nephroscope insertion. A 5-Fr ureteral catheter was inserted with nitinol guidewire with diameter of 0.025-inch and length of 150-cm as a guidance under 22.5-Fr cystoscopy with 70° lens. If ureteral catheter was correctly placed, urine flow could be observed at the distal end of the ureteral catheter. Before renal puncture, ultrasonography was used to assess the surrounding organs Figure 1(a). Secondary confirmation was done by injecting sterile water via ureteral catheter, if the ureteral catheter was correctly placed. water jet appearance and ureteral catheter itself could be visualized under ultrasound imaging Figure 1(b). Second guidewire was then inserted using cystoscopy to prevent distal expulsion of stone fragment to the ureter. Sterile water was being pumped via the ureteral catheter placed, while simultaneously using USG probe identifying the kidney. The probe was placed on the midaxillary line, parallel to the 11th rib. Skin-to-stone distance was measured, to estimate the puncture depth. Needle bracket with 17.5-G needle was attached to the USG probe and put in the longitudinal view to make it easier to control the puncture needle Figure 1(c) and (d). The successful puncture was confirmed by visualization of the needle tip in the pelvicalyceal system under ultrasound imaging Figure 2(a). Urine flow could be observed from the distal tip of the needle. Figure 2(b). A guidewire was inserted to the puncture needle; then the needle is removed while leaving the 0.035-inch J-shaped Stiff Guide Wire behind. An additional 1 cm transverse incision over the skin was performed to further facilitate Amplatz sheath insertion. Fascial dilators were inserted gradually from 8-Fr, 10-Fr, and 12-Fr, using guidewire as guidance and under ultrasound imaging Figure 3(a). Alken Metal Telescopic Dilator (6-Fr × 30-Fr) was inserted using guidewire as guidance and under ultrasound imaging Figure 3(b). The correct placement of fascial dilators and metal dilators in the pelvicalyceal system was confirmed by water flow from the tip each of the dilators inserted. A 30-Fr amplatz sheath was then inserted, followed by a 30-Fr Nephroscope with 0° lens after inner amplatz sheath had been removed. Stone was directly identified and fragmented into smaller parts using a 3.4-Fr Pneumatic Lithotriptor. The larger parts of fragmented stones that could fit the Amplatz sheath was evacuated using stone forceps, while smaller fragments were evacuated using the spooling technique. After most of the stone fragments has been removed, guidewire that placed earlier using cystoscopy was pulled out for safety reason. If any active bleeding and/or infundibulum laceration were noticed, a 6-Fr DJ stent and/or 8-Fr nephrostomy tube was placed. Using guide wire and nephroscope DJ stent and/or nephrostomy tube could be placed antegrade to secure patent urinary drainage after surgery [7].

Renal access time was defined as the time between when the ultrasound probe was introduced to the skin until the puncture was completed. A successful puncture was defined when there was no fluoroscopy performed. Tract dilatation time was defined as the time recorded between when the first fascial dilator was introduced to the skin until the removal of the inner Amplatz sheath. A stone-free evaluation was done 24 h post-PCNL. Stone-free status was defined when there was no residual stone or residual stone <4 mm for the largest diameter on the kidney-ureter-bladder plain radiography evaluation taken 1-day post-PCNL.

### 2.2 Statistical analysis

Statistical analysis was performed using SPSS V. 24 (IBM Corp., Armonk, NY, USA). Numerical data were analysed using the Kolmogorov-Smirnov test of normality and reported using mean ± standard deviation, median, and min-max value as appropriate. Categorical data were reported using percentages. One-way ANOVA, Kruskal-Wallis, and chi-square analyses were used to compare groups based on the type of data and normality of data, to ensure similar patient characteristics between treatment groups. The chi-square test was used to analyse the stone-free-rate and complication rate between the last treatment group and the control group. Line charts were used to evaluate the learning curve. A modified Clavien-Dindo classification was used for any complications recorded during the study.

## 3 Results

Table 1 summarises and compares the patient data for each treatment group. No significant differences were found among the patient groups for most variables, except preoperative stone diameter (p < 0.01), stone complexity evaluated using GUY's Stone Score (p = 0.045) and puncture approach (p < 0.01). However, statistical analysis between the five treatment groups found no statistically significant differences in preoperative stone diameter (p = 0.17) and stone complexity (p = 0.079). From the bivariate analysis, no significant difference was found in terms of ASA and clavien dindo. This shows the same characteristics between the control group and the total group so that the decrease in operating time is indeed due to differences in skills and experience not due to differences in patient characteristics.

**Table 1:** Patient characteristics.

	Total (n = 50)	Control (n = 50)	1st^b^	2nd^b^	3rd^b^	4th^b^	5th^b^	p-value
Age [mean (SD)]	51.28 (16.7)	53.3 (7.2)	54.6 (12.9)	51.2 (21)	55.2 (14.7)	49.5 (9.8)	45.9 (23.2)	0.78
Gender								0.46
Male	21 (42%)	5 (50%)	5 (50%)	3 (30%)	6 (60%)	5 (50%)	2 (20%)	
Female	29 (58%)	5 (50%)	5 (50%)	7 (70%)	4 (40%)	5 (50%)	8 (80%)	
Pre-operative Urea, mg/dL	32.05 (10.5–264.2)	41.5 (12–123)	67.8 (22–92.2)	36 (27.5–264.2)	30.2 (17.4–70.5)	33.6 (16–98.4)	27.7 (10.5–122.7)	0.29
Pre-operative Creatinine, mg/dL	1.3 (0.2–9.4)	1.1 (0.7–8.8)	2.5 (0.6–5.3)	1.2 (0.7–8.6)	1.3 (0.8–9.4)	1.2 (0.6–4.4)	1.2 (0.2–8.2)	0.67
ASA Status								
ASA I	10	9	4 (40%)	3 (30%)	4 (40%)	3 (30%)	3 (30%)	0.13
ASA II	25	20	3 (30%)	4 (40%)	3 (30%)	4 (40%)	4 (40%)	0.28
ASA III	15	21	3 (30%)	3 (30%)	3 (30%)	3 (30%)	3 (30%)	0.15
Clavien-Dindo Classification								
I								
II	29	28	5	4	4	5	6	0.88
IIIA	20	20	3	4	3	4	3	0.69
IIIB	1	2	2	2	3	1	1	0.76
IVA	0	0	0	0	0	0	0	
IVB	0	0	0	0	0	0	0	
V	0	0	0	0	0	0	0	
BMI	0	0	0	0	0	0	0	0.24
Kidney side								0.12
Left	26 (52%)	4 (40%)	2 (20%)	7 (70%)	5 (50%)	7 (70%)	3 (30%)	
Right	24 (48%)	6 (60%)	8 (80%)	3 (30%)	5 (50%)	3 (30%)	7 (70%)	
Hydronephrosis Grade								
Non	22 (44%)	6 (60%)	1 (10%)	4 (40%)	5 (50%)	6 (60%)	6 (60%)	0.21
I	1 (2%)	1 (10%)	-	-	-	-	1 (10%)	
II	3 (6%)	-	1 (10%)	-	2 (20%)	-	-	
III	11 (22%)	2 (20%)	2 (20%)	3 (30%)	1 (10%)	3 (30%)	2 (20%)	
IV	13 (26%)	1 (10%)	6 (60%)	3 (30%)	2 (20%)	1 (10%)	1 (10%)	
Largest Stone Diameter, mm	31 (20–56)	64 (40–91)	32.5 (20–56)	25 (20–50)	32.5 (25–54)	30.5 (26–40)	32.5 (30–45)	<0.01∗
GUY's Stone Score								0.045∗
Grade I	17 (34%)	1 (10%)	5 (50%)	4 (40%)	1 (10%)	2 (20%)	5 (50%)	
Grade II	4 (8%)	4 (40%)	-	-	-	1 (10%)	3 (30%)	
Grade III	22 (44%)	3 (30%)	3 (30%)	5 (50%)	8 (80%)	5 (50%)	1 (10%)	
Grade IV	7 (14%)	2 (20%)	2 (20%)	1 (10%)	1 (10%)	2 (20%)	1 (10%)	
Skin-to-stone, mm	55.8 (17.4)	-a	49.5 (9.2)	54.2 (14.2)	60.5 (25.5)	57.1 (11.3)	57.8 (7)	0.69^c^
History of past kidney surgery	19 (38%)	2 (20%)	6 (60%)	4 (40%)	6 (60%)	2 (20%)	1 (10%)	0.07
Puncture Approach								<0.01∗
Superior Calyx	13 (26%)	1 (10%)	3 (30%)	1 (10%)	7 (70%)	-	2 (20%)	
Medial Calyx	18 (36%)	-	-	4 (40%)	3 (30%)	5 (50%)	6 (60%)	
Inferior Calyx	19 (38%)	9 (90%)	7 (70%)	5 (50%)	-	5 (50%)	2 (20%)	

∗Statistically significant in overall comparison between control and treatment groups.

a Skin-to-stone distance is not measured in supine fluoroscopy percutaneous nephrolithotomy.

b represents treatment group based on the timing of surgery, each group consists of ten patients.

c comparison between each treatment group.

**Figure 4:**
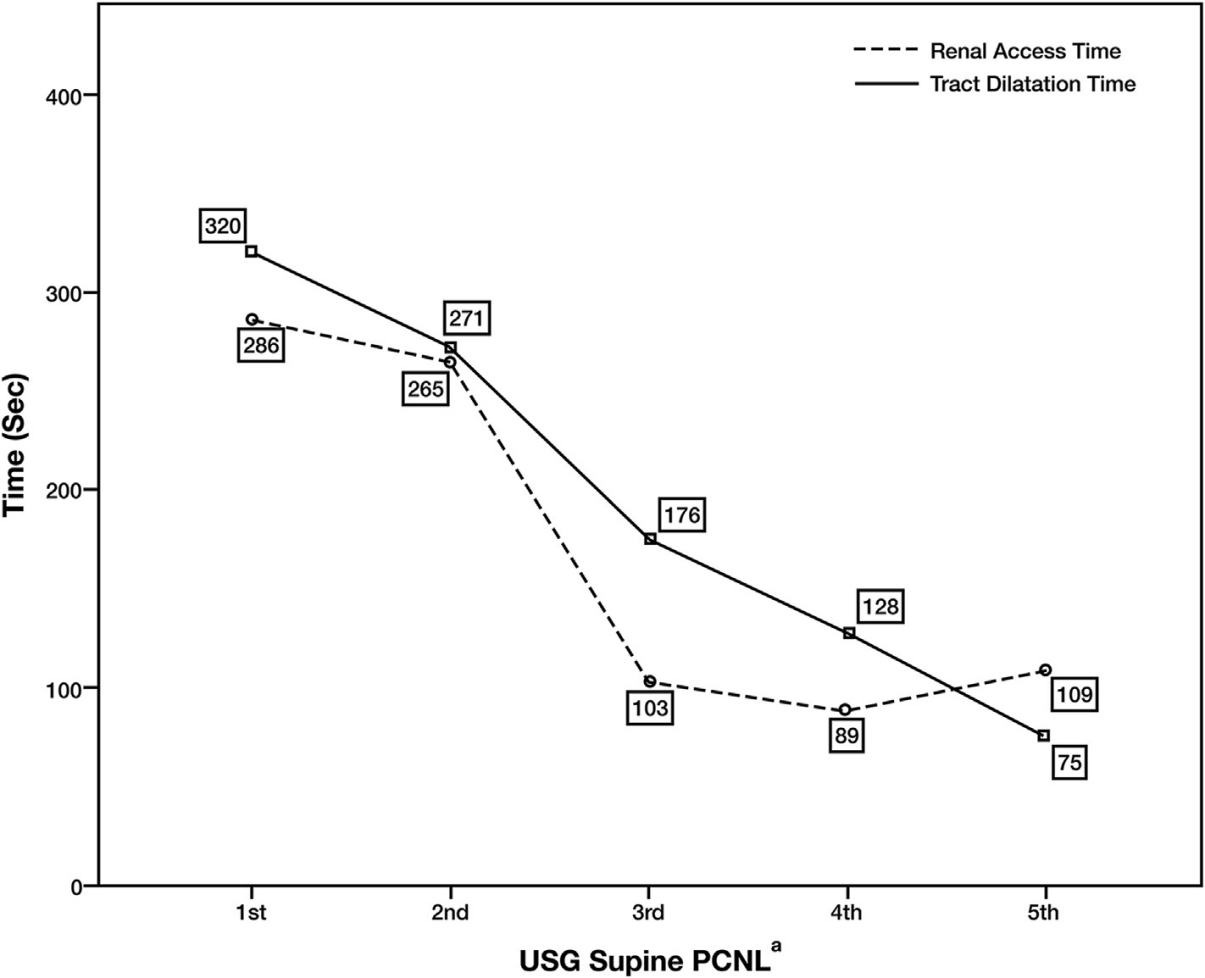
Renal access time and tract dilatation time. (a) Each of the x-axis markers (1st, 2nd, 3rd, 4th, and 5th) represents the treatment groups, each of which consists of ten patients based on the timing of surgery.

Renal access time is shown in Figure 4. The median renal access time fluctuated between 89 and 286 s. The median renal access time peaked in the 1st treatment group and gradually decreased over time until it reached the lowest value of 89 s in the 4th treatment group. Renal access time was lower after 20 PCNL procedures. However, statistical analysis using the Kruskal-Wallis test showed no significant difference between the five treatment groups (p = 0.259).

Median tract dilatation time is shown in Figure 4. The lowest tract dilatation time occurred in the 5th treatment group with a value of 75 s, while the highest time was seen in the 1st treatment group. Tract dilatation time was lower after 20 PCNL procedures, and the trend line continued to decrease as the surgeon gained more experience. Statistical analysis using the Kruskal-Wallis test showed a significant statistical difference between the five treatment groups (p < 0.001).

**Figure 5:**
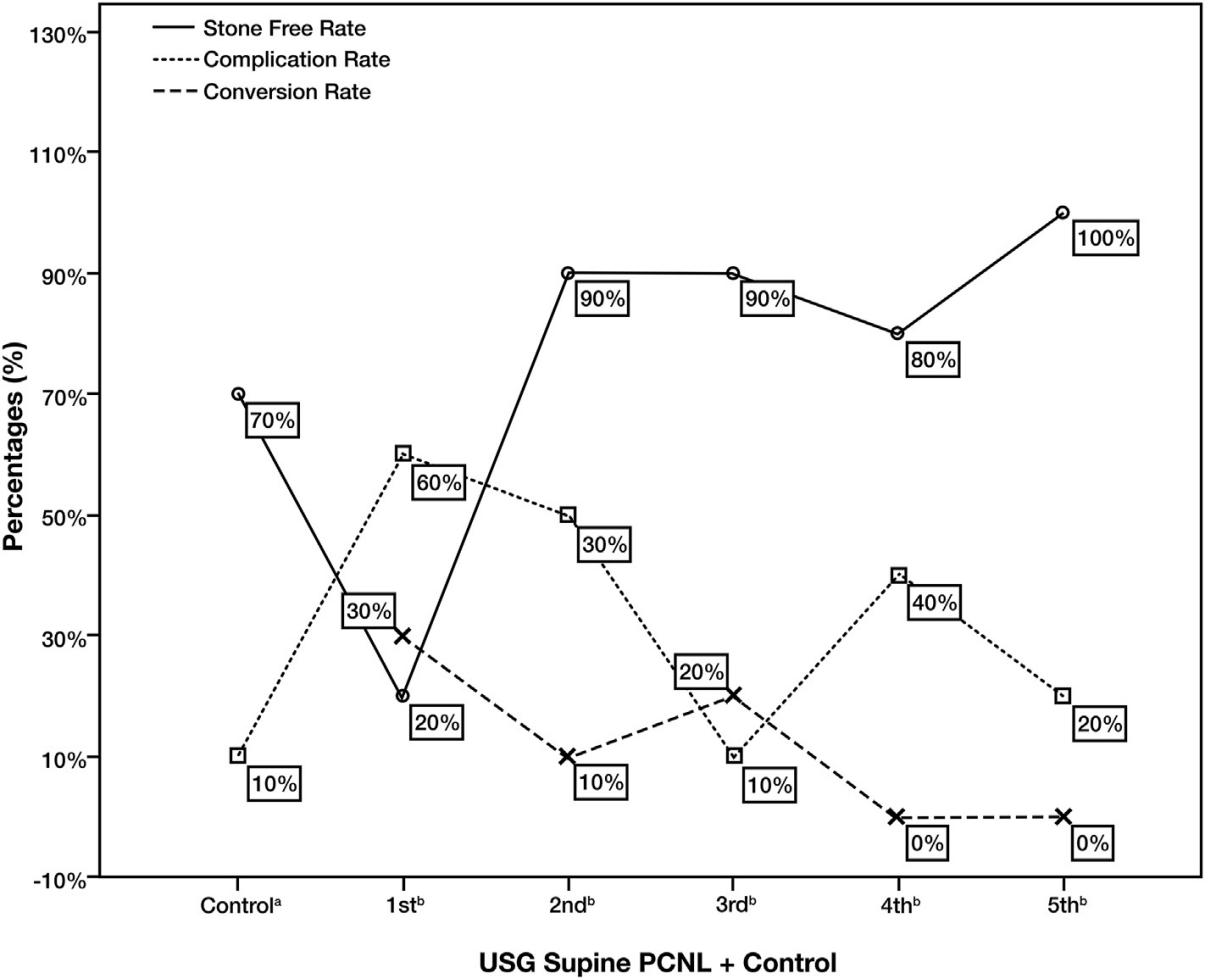
Stone-free rate, conversion rate, and complication rate. (a) The control groups consist of ten patients who underwent supine PCNL with fluoroscopy; (b) represents the treatment group, which consists of ten patients who underwent USG supine PCNL based on the timing of surgery.

The conversion rate from USG to fluoroscopic guided PCNL is shown in Figure 5. In the first ten cases, only 30% converted to fluoroscopy. As the surgeon gained more experience, the conversion rate decreased. However, the conversion rate was still fluctuating in the 3rd and 4th groups. Despite the lower conversion rate after 30 patients, statistical analysis showed no significant improvement between the five treatment groups (p = 0.169).

The stone-free status rate is shown in Figure 5. The control group had a larger median stone diameter than the treatment group because the surgeon could treat larger stones using fluoroscopy. However, when comparing the last treatment group with the control group, there was no significant difference in terms of stone-free rate (100% vs. 70%, p = 0.21). This is our first experience in performing USG Guided PCNL in Indonesia, therefore we select patients with a relatively simple urolithiasis to be treated using USG Guided PCNL technique. We avoid patients with larger stone to prevent more than one puncture. We avoid larger stone to prevent more than one punction. As for the evaluation between treatment groups, only two patients were categorised as stone-free in the 1st treatment group. The stone-free rate was greatly improved after 20 PCNL procedures. As shown in the graph, there was a continued improvement as the surgeon gained more experience. Despite one decrease, there was significant improvement between the five treatment groups (p < 0.001).

The complication rate in the control group was low at baseline due to the familiarity and experience with the technique. However, there were no significant differences between the last treatment group and the control group regarding the complication rate (p = 1.0). The complication rates decreased over time, especially after 20 PCNL procedures (Figure 5). However, the difference in complication rates over time did not reach statistical significance (p = 0.113). There were no major complications based on the modified Clavien-Dindo classification of surgical complications. Reported complications were postoperative fever (10%), which was treated with acetaminophen, and the need for a blood transfusion (26%), this moderately high number of transfusion-demand was due to the preexisting anemia before procedure. Our finding in terms of transfusion rate was higher than the results reported by Rosette et al., that was 5% [8]. This is our first experience using guided USG therefore the complication of bleeding was also hard to be controlled. As the surgeon becomes more experience  d the intraoperative bleeding was also decreased. Moreover majority of the patients have low preoperative hemoglobin. Therefore the higher transfusion rate compared to the other literature is associated with first experience and low preoperative hemoglobin.

## 4 Discussion

This study aimed to evaluate the learning curve of USG supine PCNL using the Alken metal telescopic dilator by a single urologist. The results indicate that competency is achievable after 20 PCNL procedures.

PCNL is the recommended treatment for large renal stones (>20 mm) [1, 2]. Even though PCNL is categorised as a minimal-invasive technique, it still poses a risk of complications such as renal pelvis perforation (3.4%), hydrothorax (1.8%), and significant bleeding (7.8%) [7, 8]. However, major complications decline as more surgeons practice PCNL, and advancements in imaging modalities allow for thorough preoperative evaluation of surrounding structures [4]. Other studies showed that the most common complications reported for USG supine PCNL were fever and need for blood transfusion [1, 6, 8, 9, 10, 11, 12, 13, 14]. To gain access to the pelvicalyceal system, surgeons have relied on fluoroscopy. However, this technique brings a risk of radiation exposure to the surgeons, operating theatre nurses, technicians, and the patients themselves. Ultrasonography eliminates the radiation risk. It is an effective tool for locating the stone, especially for stones with non-opaque characteristics that are not visible using fluoroscopy, and the organs adjacent to the PCNL tract, thus preventing organ damage [1, 6].

Various studies have concluded that USG supine PCNL had similar efficacy to fluoroscopy (fluoroscopy-guided PCNL) in terms of stone-free rate, length of hospitalisation, and complication rate [6, 8, 9, 10, 11, 12, 13]; however, these studies commonly include balloon dilators. This study adds to the literature by focusing on USG PCNL with the Alken metal telescopic dilator. The Alken metal telescopic dilator was used due to its reusable properties, which could lower the cost of PCNL. In terms of patient position, meta-analysis has shown that the supine position has the same efficacy and safety as the prone position [15]. The supine position for PCNL provides benefits such as eliminating the need for repositioning, enabling simultaneous retrograde cystoscopy insertion without repeated asepsis and antisepsis procedures, allowing the surgeon to observe the water flow from the distal tip of the Alken metal telescopic dilator during the dilatation of the tract (which can only be done in the supine position), permitting the surgeon to work on the lower intrarenal pressure, minimum risk of colonic puncture, and easier management of cardiac and pulmonary emergencies.

In the present study, improvement in conversion rate was seen after 20 PCNL procedures were performed. However, there was a minor setback possibly associated with the surgeon's interest in treating more complex cases, such as larger stone size and non-hydronephrosis patients. Hydronephrosis may assist the surgeon in reaching the pelvicalyceal system as it allows for a shorter puncture distance and makes it easier to differentiate the kidney anatomy. A similar result was shown in a systematic review in which puncture success rate was about 30% in the first 20 cases and rose to 75% starting at the 21st case, finally reaching 100% at the end of the study [10].

Renal access time improved after 20 PCNL procedures. This was due to the surgeon's increased familiarity with ultrasound kidney visualisation and instrumentation, experience identifying adjacent organs, and improved dexterity. However, a minor decline in renal access time appeared in the 4th group, presumably related to the fact that it was the group with the lowest hydronephrosis status. Therefore, the surgeon had to re-adjust from the normal ultrasound kidney appearance to the hydronephrosis kidney, which has a distinguished needle appearance reaching the pelvicalyceal system. This may also have caused the minor setback shown in the stone-free-rate decline in the 4th group.

The Seldinger technique was used for the dilatation tract. The surgeon evaluated the water coming from the tip of the dilator to confirm that it had been inserted in the correct position. With more experience and improved dexterity, the tract dilatation time markedly reduced. The present results showed a significant reduction in tract dilatation time (p < 0.001), with the most improvement seen after 20 PCNLs were performed. A similar result was reported by Iordache et al., where inexperienced surgeons needed 60 PCNLs, and experienced surgeons needed 20 PCNLs to achieve the same level of competency [10].

Stone-free status is still considered as the most important indicator of competency. Our study shows similar results to the study by Song et al., which concluded that a surgeon could completely remove all the stones even while still learning the procedure [1]. Despite progress overall, the stone-free-rate average in this study varied from 60 to 93.3%, showing a p-value of 0.714, while our study showed a p-value of <0.01.

In total, the complication rate was 36% for our study. Using modified Clavien-Dindo classifications, most of the complications involved the need for a blood transfusion because of active bleeding in the lacerated area of the infundibulum. Moreover, this was due to slightly lower mean pre-operative haemoglobin levels, which were 12.03 ± 2.1, leading to post-operative transfusions, especially in the first ten cases. As familiarity with ultrasound improved, the complication rate was lower. This compares with a complication rate of 18% that was shown by Ng et al. [12], where angioembolisation was needed. The median amount of transfusion was 234 mL (200–647 mL). In 10% of the complications, the patients experienced a fever that was easily managed with acetaminophen postoperatively.

This study was limited in that it describes the PCNL learning curve from a single surgeon. Ideally, further studies of the learning curve with other experienced surgeons in the field of endourology, especially with fluoroscopic PCNL, should be done so that an average number of USG PCNLs could be calculated. Thus, more reliable data could be provided for other practising surgeons.

USG supine PCNL using an Alken metal telescopic dilator is a safe and effective procedure for experienced surgeons to perform. Basic competency is achievable after performing a minimum of 20 PCNL procedures, and further improvement in outcomes can be achieved after 40 procedures with minimal complication rates of mild severity. Small sample size, a lack of power analysis, and a retrospective design may be additional limitations of the study.

## Declarations

### Author contribution statement

Ponco Birowo, MD, PhD: Conceived and designed the experiments; Performed the experiments.

Reginald Rustandi, MD: Analyzed and interpreted the data; Contributed reagents, materials, analysis tools or data; Wrote the paper.

Putu Angga Risky Raharja, MD; Harun Wijanarko Putra, MD: Analyzed and interpreted the data; Contributed reagents, materials, analysis tools or data.

Nur Rasyid, MD, PhD; Widi Atmoko, MD: Conceived and designed the experiments; Contributed reagents, materials, analysis tools or data.

### Funding statement

Dr Ponco Birowo was supported by 10.13039/501100006378Universitas Indonesia [Grant Number NKB-4180/UN2. RST/HKP.05.00/2020).].

### Data availability statement

Data will be made available on request.

### Declaration of interest's statement

The authors declare no competing interests.

### Additional information

No additional information is available for this paper.
